# Postoperative Adjuvant Chemotherapy Improved the Prognosis in Locally Advanced Cervical Cancer Patients With Optimal Response to Neoadjuvant Chemotherapy

**DOI:** 10.3389/fonc.2020.608333

**Published:** 2020-12-07

**Authors:** Xiaojie Feng, Hongmin Chen, Lei Li, Ling Gao, Li Wang, Xupeng Bai

**Affiliations:** ^1^ Department of Gynecologic Oncology, the Affiliated Cancer Hospital of Zhengzhou University, Zhengzhou, China; ^2^ Cancer Care Centre, St George Hospital, Sydney, NSW, Australia; ^3^ St George and Sutherland Clinical School, Faculty of Medicine, UNSW Sydney, NSW, Australia

**Keywords:** cervical cancer, neoadjuvant chemotherapy, optimal response, survival, toxicity

## Abstract

**Background:**

Few studies investigated the effectiveness of adjuvant chemotherapy (ACT) in patients with optimal response to neoadjuvant chemotherapy (NACT), and an optimal number of treatment cycles for these patients remains unknown.

**Methods:**

A total of 261 Chinese patients with FIGO stage IB2-IIB cervical cancer who obtained an optimal response to NACT were included after radical surgery, and the disease-free survival (DFS) and overall survival (OS) of these patients treated with different cycles of postoperative ACT were compared using the Log-rank test and multivariate analysis.

**Results:**

We found that the prognosis of optimal responders treated with postoperative ACT was significantly better than those without further adjuvant therapy. The multivariate analysis showed that postoperative ACT was an independent prognostic factor for DFS. However, there was no significant difference in the DFS and OS between patients who had three cycles of ACT and those with six cycles. Further analysis revealed a significant association of six cycles of ACT with the risk of leukopenia, nausea/vomiting, and rash.

**Conclusion:**

Our data suggest that additional three cycles of ACT after surgery may improve the clinical outcome of optimal responders in terms of DFS, OS, and drug toxicity.

## Introduction

Cervical cancer is the second most common gynecologic cancer in developing countries. The estimated annual incidence and mortality of cervical cancer showed an income-dependent distribution pattern with the greatest disease burden in sub-Saharan Africa, Southeast Asia, and Latin America due to the poor access to screening and treatment (1). Almost 530,000 new cases are diagnosed worldwide each year, and approximately 270,000 women die of cervical cancer annually (2).

Neoadjuvant chemotherapy (NACT) followed by radical surgery (RS) is considered as a valid alternative for locally advanced cervical cancer and utilized in many countries. The sequence contributes to a substantial improvement towards disease control and survival for stage IB2–IIB tumors as compared to the radiotherapy (RT) alone (3). Nevertheless, there were still around 20% to 30% of patients treated with NACT+RS suffering from pelvic and/or extra-pelvic recurrence in 5 years, and long-term overall survival (OS) is still unsatisfactory (4). For these reasons, postoperative adjuvant chemotherapy (ACT) has been employed with the intention of radical cure (5). It was reported that additional three to six cycles of platinum-based ACT were of benefit for patients after NACT+RS in terms of 5-year OS and disease-free survival (DFS) (6–10). However, few studies investigated the effectiveness of postoperative ACT in patients with optimal response to NACT, and an optimal number of treatment cycles for these patients remains unknown.

In this study, we aim to evaluate the effectiveness of postoperative ACT in cervical cancer patients with optimal response to NACT. Moreover, three and six cycles of ACT were compared in terms of OS, DFS, and drug toxicity. These data may provide clinical evidence for a better practice of ACT in treating cervical cancer.

## Materials and Methods

### Study Population

This is a single-institution retrospective study, which was approved by the Medical Ethics Committee in the Affiliated Cancer Hospital of Zhengzhou University (Approval No.2017416) in accordance with the 1964 Helsinki declaration and its later amendments and with the Chinese laws and regulations. Informed consent is not required for the study. Between April 2009 and September 2016, patients with FIGO stage IB2-IIB cervical cancer referred to the Department of Gynecologic Oncology were included in this study. The patient inclusion criteria included: 1) female with squamous cell, adenosquamous, or adenocarcinoma of the cervix; 2) age between 18 and 75 years; 3) having normal hepatorenal, cardiac, and respiratory functions. The exclusion criteria included: 1) severe other organ injuries; 2) a history of any disease that may exert influence on this study and other unacceptable diseases, including abnormal bone marrow function, uncontrolled infection, diabetes mellitus, acquired immune deficiency syndrome, substance dependence, and neurological or mental diseases; 3) pregnancy.

### Adjuvant Chemotherapy

All patients included in the study received NACT that is comprised of two cycles of the scheme (cisplatin 75 mg/m² and paclitaxel 175 mg/m² at 3-week intervals). Then, patients were assessed for the treatment response according to the WHO criteria (the WHO handbook for reporting results of cancer treatment: http://www.who.int/iris/handle/10665/37200). Patients with stable or progressive disease were not suitable for surgery, and thus they were excluded from this study and sent to RT.

Included patients underwent bilateral systematic pelvic lymph node dissection, Piver-Rutledge-Smith type II or type III radical hysterectomy, and bilateral salpingo-oophorectomy. Aortic lymphadenectomy was performed in patients with pelvic node disease or bulky aortic nodes.

After surgery, the pathological evaluation of surgical specimens was performed to determine the response to NACT. Patients who had positive nodes, positive surgical margins, or vaginal margins less than 0.5 mm were excluded from the study and submitted to RT. Complete response was defined as the complete disappearance of the tumor in the cervix with negative nodes. Optimal partial response was defined as a persistent residual disease with < 3 mm stromal invasion, including *in situ* carcinoma on the surgical specimen and negative lymphatic metastasis. Overall optimal response was defined as the sum of complete and optimal partial response. Patients who did not achieve optimal response were excluded from this study.

Thereafter, patients received three to six cycles of ACT (same regime as NACT) at 3-week intervals beginning 3 to 4 weeks after surgery. Chemotherapeutic toxicity was recorded and graded according to the WHO criteria (the WHO handbook for reporting results of cancer treatment: http://www.who.int/iris/handle/10665/37200). Treatment was delayed, or dosage was decreased in the case of G3 toxicity. Treatment was discontinued in the case of G4 or life-threatening toxicity. Cisplatin was replaced with carboplatin and paclitaxel was replaced with topotecan if severe hypersensitivity reaction occurred.

### Follow-Up Surveys

Real-time demographic and clinical data were obtained from the electronic medical record system at the Department of Gynecologic Oncology. At the end of ACT, all patients were followed up every 3 to 4 months for the first 2 years, then every 6 months for the following 3 years, and annually thereafter. Each follow-up included an essential interview, physical examination, and vaginal cytology for identifying lower genital tract tumors. Computed tomography or magnetic resonance imaging was used to examine abdominal and pelvic regions. The biopsy was performed to evaluate suspicious cases of recurrent cancer. Cancer recurrence evidence was defined as either regional recurrence or distant metastasis.

### Statistical Analysis

The DFS was calculated from the date of the first cycle of NACT to the date of documented evidence of recurrence, while the OS was defined as the time from the first cycle of NACT to death or last follow-up. Survival curves were generated using the Kaplan-Meier method and compared by the Log-rank test. Cox proportional hazard model was employed for multivariate analysis. Other analyses were done using the Chi-Square test and Fisher test. Statistical significance was set at *P* < 0.05. All comparisons were performed using the SPSS (v23.0, IBM Analysis, USA).

## Results

### The Effect of Postoperative Adjuvant Chemotherapy on the Prognosis in Patients With Optimal Response to Neoadjuvant Chemotherapy

From April 2009 to September 2016, 4373 patients were assessed according to the inclusion and exclusion criteria. 1654 patients received NACT+RS. Thereinto, 261 (15.8%) patients who achieved an optimal response were included in the analysis. The pathological response was complete in 61 patients, optimal partial in 200. 152 (58.2%) patients underwent Piver-Rutledge-Smith type III radical hysterectomy, while 109 (41.8%) patients underwent Piver-Rutledge-Smith type II radical hysterectomy.

After surgery, 60 (23.0%) patients were not treated with further ACT; 101 (38.7%) patients received three cycles of ACT; 16 (6.1%) patients underwent four cycles of ACT; 22 (8.4%) patients had five cycles of ACT; 62 (23.8%) patients underwent six cycles of ACT. There were 15 (5.8%) and 6 (2.3%) patients showing hypersensitivity reactions to cisplatin and paclitaxel, respectively, and thus the drugs were replaced as described in the method.

At the time of the analysis, 17 (6.5%) out of the 261 patients relapsed after a median time of 15.8 months (range, 5.5 to 43.3 months); 9 (3.5%) out of the 261 patients died after a median time of 22.9 months (range, 19.3 to 41.3 months). As shown in **Table 1**, the recurrent disease was pelvic in 8 (3.1%) cases, extra-pelvic (aortic or distant) in 7 (2.7%) cases, and both pelvic and extra-pelvic in 2 (0.8%) cases. According to the cycle number of ACT, tumor relapsed in 8 (13.3%) out of the 60 patients without further therapy (5 pelvic recurrences [2 died], and 2 extrapelvic recurrences [died], and 1 pelvic + extrapelvic recurrence [died]), 5 (5.0%) out of the 101 patients with three cycles (2 pelvic recurrences, 2 extra-pelvic recurrences [1 died], and 1 pelvic + extra-pelvic recurrence [died]), 1 (6.3%) out of the 16 patients with four cycles (1 pelvic recurrence [died]), 1 (4.6%) out of the 22 patients with five cycles (1 extra-pelvic recurrence), and 2 (3.2%) out of the 62 patients with six cycles (1 pelvic recurrence and 1 extra-pelvic recurrences [died]) (**Table 1**).

**Table 1:** Recurrence and death rate in optimal responders according to the cycle number of postoperative ACT.

Cycle number of ACT	Pts	Recurrence and death profile			Overall	
		P	EX	P + EX	Recurrence	Death
0 cycles	60	5 (2 died)	2 (died)	1 (died)	8 (13.3%)	5 (8.3%)
3 cycles	101	2	2 (1 died)	1 (died)	5 (5.0%)	2 (2.0%)
4 cycles	16	0	1 (died)	0	1 (6.3%)	1 (6.3%)
5 cycles	22	0	1	0	1 (4.5%)	0 (0.0%)
6 cycles	62	1	1 (died)	0	2 (3.2%)	1 (1.6%)

ACT, adjuvant chemotherapy; Pts, patients; P, pelvic; EX, extra-pelvic.

**Figure 1:**
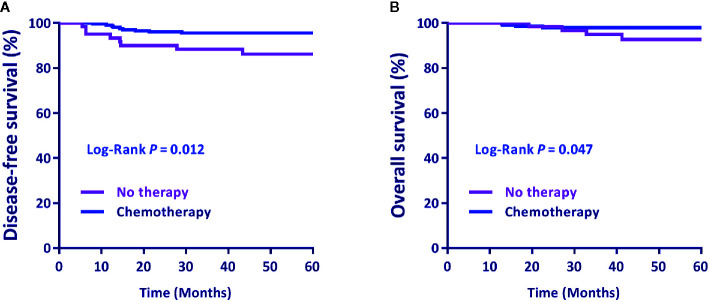
**(A)** Disease-free survival (DFS) and **(B)** overall survival (OS) by postoperative treatment. Differences in DFS and OS by treatment were evaluated using the Log-rank test. *P* < 0.05 was statistically significant.

The DFS and OS of patients treated with postoperative ACT were significantly better than patients without postoperative therapy (*P* =0.012, HR = 3.162, 95% CI = 1.002–5.971 and *P* = 0.047, HR = 3.207, 95% CI = 1.080–7.782, respectively) (**Figures 1A, B**).

Multivariate analysis on the whole series using the Cox proportional hazard model showed that the DFS and OS rate of patients with squamous cell cancer were significantly higher than those with adenosquamous (*P* = 0.0007, HR = 1.834, 95% CI = 1.285–2.322 and *P*=0.0002, HR=2.057, 95%CI=1.182–2.937, respectively) (**Table 2**). Further analysis indicated that the DFS of patients who received postoperative ACT was better than those who did not (*P* = 0.033, HR = 1.740, 95%CI = 1.103–2.369), whereas the difference in the OS between the two groups became non-significant after multivariate correction (*P* = 0.097) (**Table 2**).

**Table 2:** Multivariate analysis of variables predictive of the DFS and OS in optimal responders.

Variables	Pts	5-year DFS	P value	5-year OS	*P*-value
Age					
≤46	158	93.7%	0.886	96.8%	0.896
>46	103	91.9%		95.1%	
Histology					
Squamous cell	258	93.5%	0.0007*	96.8%	0.0002*
Adenosquamous	3	33.3%		33.3%	
Adenocarcinoma	0	–		–	
FIGO stage					
IB2	177	93.2%	0.834	97.2%	0.745
IIA	84	91.6%		93.0%	
IIB	0	–		–	
Grade					
G1	54	94.4%	0.769	96.3%	0.906
G2	68	94.1%		95.5%	
G3	139	91.4%		96.4%	
ACT					
Yes	201	95.5%	0.033*	98.0%	0.097
No	60	86.1%		92.7%	

DFS, disease-free survival; OS, overall survival, ACT, adjuvant chemotherapy; Pts, patients; FIGO, International Federation of Gynecology and Obstetrics.

*P < 0.05 was statistically significant.

### Comparison between Three and Six Cycles of Postoperative Adjuvant Chemotherapy in Optimal Responders in Terms of Prognosis and Toxicity

Next, patients with three or six cycles of ACT were selected for the following analysis to investigate the effect of the cycle number of ACT on the prognosis of optimal responders. The potential variables that may affect the efficacy of ACT in the present study were assessed by univariate analysis, and there was no statistically significant difference between the two groups (**Table 3**).

**Table 3:** Characteristics of optimal responders treated with 3 or 6 cycles of postoperative ACT.

Variables	3 cycles (n = 101)	6 cycles (n = 62)	*P* value
Age			
≤46	56 (55.4%)	37 (59.7%)	0.628
>46	45 (44.6%)	25 (40.3%)	
Histology			
Squamous cell	101 (100.0%)	61 (98.4%)	0.380
Adenosquamous	0 (0.0%)	1 (1.6%)	
FIGO stage			
IB2	64 (63.4%)	40 (64.5%)	1.000
IIA	37 (36.6%)	22 (35.5%)	
Grade			
G1	14 (13.9%)	9 (14.5%)	0.938
G2	37 (36.6%)	21 (33.9%)	
G3	50 (49.5%)	32 (51.6%)	

FIGO, International Federation of Gynecology and Obstetrics.

**Figure 2:**
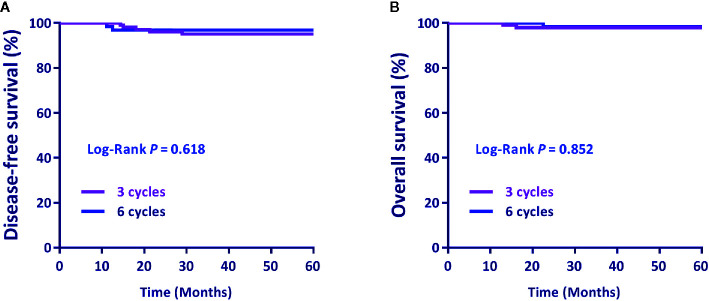
**(A)** Disease-free survival (DFS) and **(B)** overall survival (OS) by the cycle number of postoperative adjuvant chemotherapy. Differences in DFS and OS by treatment were evaluated using the Log-rank test.

There was no significant difference in the DFS and OS between patients who had three cycles of ACT and those who received six cycles (*P*=0.618 and *P*=0.852, respectively) (**Figures 2A, B**).

The toxicity profile among patients with three or six cycles of ACT is shown in **Table 4**. The most frequent adverse reaction was leukopenia and then nausea/vomiting. Specifically, leukopenia occurred in 67.7% of patients with six cycles of ACT and in 39.7% of patients with three cycles; nausea/vomiting occurred in 59.7% of patients with six cycles of ACT and in 36.6% of patients with three cycles. Analysis of these data showed statistically significant differences between the two groups in leukopenia (*P* = 0.003, RR = 2.683, 95% CI = 1.200–5.998) and nausea/vomiting (*P* = 0.017, RR = 1.314, 95% CI = 1.176–2.222). Furthermore, the rash incidence also showed a significant difference between the two groups (*P*=0.023, RR=3.889, 95%CI=1.073–14.097). In contrast, there was no significant difference in thrombocytopenia (*P*=0.413), anemia (*P*=0.505), absolute neutrophil (*P*=1.000), peripheral sensory neuropathy (*P*=0.329), and hepatotoxicity/nephrotoxicity (*P*=1.000) between the two groups.

**Table 4:** The associations of postoperative ACT with toxicity in optimal responders.

Toxicity	3 cycles (n = 101)	6 cycles (n = 62)	*P* value
Leukopenia			
G3	35 (34.7%)	24 (38.7%)	0.003*
G4	5 (5.0%)	18 (29.0%)	
Thrombocytopenia			
G3	7 (6.9%)	4 (6.5%)	0.413
G4	5 (5.0%)	8 (12.9%)	
Anemia			
G3	11 (10.9%)	7 (11.3%)	0.505
G4	8 (7.9%)	10 (16.1%)	
Absolute neutrophil			
G3	3 (3.0%)	10 (16.1%)	1.000
G4	1 (1.0%)	2 (3.2%)	
Nausea and vomiting			
G3	28 (27.7%)	17 (27.4%)	0.017*
G4	9 (8.9%)	20 (32.3%)	
Peripheral sensory neuropathy			
G3	2 (2.0%)	7 (11.3%)	0.329
G4	3 (3.0%)	3 (4.8%)	
Hepatotoxicity/nephrotoxicity			
G1	2 (2.0%)	4 (6.5%)	1.000
G2	1 (1.0%)	3 (4.8%)	
Dermatology (rash)			
G1	7 (6.9%)	2 (3.2%)	0.023*
G2	2 (2.0%)	8 (12.9%)	

*P < 0.05 was statistically significant.

## Discussion

The clinical practice of postoperative ACT for locally advanced cervical cancer patients with optimal response to NACT is less verified. This is because no pathological risk factor can be used to evaluate the risk of recurrence and death in these optimal responders, and no standard guideline can be referred to decide the optimized course of ACT. Many studies reported that the survival time of optimal pathological responders was significantly longer than that of non-optimal responders, considering optimal response as a favorable prognostic factor and surrogate endpoint for survival (4, 5, 11, 12), which may explain why the postoperative management for these patients did not gain enough attention.

In this study, we noticed that the recurrence rate in optimal responders without postoperative therapy (13.3%) is higher than European studies (range, 10.9%–11.1%) (4, 13). The possible explanation is the different cycle number of NACT because, in Asian countries, such as China and Japan, two cycles of platinum-based NACT were widely accepted for patients (11, 12, 14–16), while three cycles were widely used in European countries (4, 7, 17).

The response rate to neoadjuvant therapy varies among different studies because of the different regimes. Previous studies indicated that the complete response rate of neoadjuvant radiochemotherapy ranged from 40% to 70% (18, 19), which is much higher than NACT alone. Although most optimal responders survived without evidence of recurrence for a lifetime, the long-term recurrent cases did exist in these patients. Indeed, a small minority of optimal responders carried invisible distant micro-metastasis that might explain the tumor relapse after several years (13). Compared with adjuvant RT that shows an effective local recurrence control, ACT has an advantage in treating extra-pelvic metastasis (20). Consistently, in our study, 5.0% of patients (3 out of 60) without therapy experienced extra-pelvic recurrence as compared to 3.0% of patients with ACT (6 out of 201), suggestive of the importance of additional ACT in eliminating the potential metastatic lesions. More importantly, patients with ACT showed higher DFS and OS rate as compared to those without further therapy, indicating that ACT can still improve the prognosis even if they obtained an optimal response to NACT. Landoni (13) reported that there was no significant difference in the risk of recurrence between 0 and 2 cycles of ACT in overall optimal responders (11.1% vs 11.1%). It is complicated to compare them to our results because the sample size and the cycle number of ACT they used are less than ours.

Some studies indicated that postoperative ACT could be given according to the number of high-risk and/or intermediate-risk factors that patients have (6, 21). However, no risk or reliable prognostic factor was identified in optimal responders after surgery for choosing the optimized cycle number of ACT. As a result, the duration of ACT for these patients was performed according to the institutional experience, ranging from 0 to 6 cycles. In our experience, three to six cycles of CT were often used for patients based on individual tolerance. In this study, we found that postoperative ACT was an independent prognostic factor for optimal responders, whereas there was no significant difference in the DFS and OS between three and six cycles.

Besides, we observed that the cancer subtype in most optimal responders was squamous cell types, suggesting that squamous cell carcinomas are more sensitive to CT than adenosquamous cancer and adenocarcinoma. This is consistent with a study indicating that the chemotherapeutic response was more favorable in squamous cell carcinomas (87%) than that in adenocarcinomas (38%) (*P*=0.01) (22). Notably, no patient with stage IIB was found to achieve optimal response in the current study because of the finding of high-risk and/or intermediate risk factors after surgery, such as positive nodes and deep muscle invasion.

On the other hand, three cycles of postoperative ACT showed a favorable toxicity profile as compared to six cycles. A significantly higher incidence of leukopenia and nausea/vomiting was observed in the six-cycle group. Our data of hematological toxicity are comparable to other studies regarding patients submitted to 6 courses of platinum-based ACT who showed 9.2% thrombocytopenia, 23.2% anemia, and 15.6% febrile neutropenia, whereas the incidence of nausea/vomiting and neurotoxicity in our study is much higher than other studies showing 17.9% vomiting and 3.3% neurotoxicity (23). The possible explanation for this might be the racial difference. In addition, six cycles of ACT also caused an increase in the incidence of G2 rash. These data suggest that three courses of ACT are more tolerated with acceptable adverse reactions, which may be appropriate for patients to both comply with the following therapy and improve survival quality.

In summary, the findings from this study indicate that postoperative ACT can help further improve the clinical outcome of optimal responders. Compared with six cycles, three cycles of postoperative ACT could be of benefit for these patients in terms of prognosis and drug toxicity.

## Data Availability Statement

The raw data supporting the conclusions of this article will be made available by the authors, without undue reservation.

## Ethics Statement

The studies involving human participants were reviewed and approved by the Medical Ethics Committee in the Affiliated Cancer Hospital of Zhengzhou University. Written informed consent for participation was not required for this study in accordance with the national legislation and the institutional requirements.

## Author Contributions

XF conceived and designed the study. XF performed literature research and clinical data collection. HC, LL, LG, and LW assisted with the study. XF and XB performed data analysis and manuscript preparation. All authors contributed to the article and approved the submitted version.

## Funding

This study was supported by the Project of Science and Technology Department of Henan Province (192102310066). The authors would also like to acknowledge the funding support from UNSW Sydney and China Scholarship Council.

## Conflict of Interest

The authors declare that the research was conducted in the absence of any commercial or financial relationships that could be construed as a potential conflict of interest.
